# Blood ACE Activity Is Significantly Lowered in Psoriasis Compared to Atopic Dermatitis: A Small Pilot Study in Young Males

**DOI:** 10.1007/s11596-026-00232-6

**Published:** 2026-07-20

**Authors:** Vivien Osterhus, Markus Böhm, Simone König

**Affiliations:** 1 Service Unit Proteomics, Medical Faculty, University of Münster, Münster, 48149 Germany; 2 Department of Dermatology, University of Münster, Münster, 48149 Germany

**Keywords:** Angiotensin-converting enzyme, Renin-angiotensin system, Psoriasis vulgaris, Atopic dermatitis, Bradykinin, Carboxypeptidase N, Autoimmune skin disease, Neuropeptide reporter assay, Thin-layer chromatography

## Abstract

**Purpose:**

Emerging evidence suggests the involvement of the renin-angiotensin system (RAS) in the pathogenesis and progression of autoimmune dermatological diseases. In a small exploratory study, we investigated angiotensin-converting enzyme (ACE) activity in blood obtained from male probands with psoriasis vulgaris (n = 4) and atopic dermatitis (AD, n = 5).

**Methods:**

The degradation capacity of dabsylated synthetic bradykinin (DBK) with and without inhibition was determined using a thin-layer chromatography (TLC)-based neuropeptide reporter assay.

**Results:**

We observed a significantly reduced capacity for cleavage of DBK in psoriasis patients compared with that in AD patients and controls. In patient samples, the variation in the measured values was generally greater than that in healthy controls. We could not confirm the increased ACE activity in the circulation in psoriasis patients reported by others, likely because of different study designs and detection methods. We did not include samples of female patients and focused on younger men to avoid hormonal effects and minimize age-related factors.

**Conclusion:**

This preliminary study of the hypothesis-generating nature lacks power, but it certainly adds a question to the current view of the role of ACE in psoriasis. Treatments targeting specific components of the RAS could ameliorate inflammatory responses; thus, research in this area is becoming increasingly important.

**Supplementary Information:**

The online version contains supplementary material available at 10.1007/s11596-026-00232-6.

## Introduction

Emerging evidence suggests the involvement of the renin-angiotensin system (RAS) in the pathogenesis and progression of autoimmune dermatological diseases, as components of the RAS, such as angiotensin receptor 1 (AT1R) and angiotensin II (Ang II), have been detected in cutaneous and subcutaneous layers [1]. Tissues such as the skin may have their own localized RAS [1]. The RAS is known to be important for controlling inflammation and immune responses. Ang II acts on AT1R and AT2R; disturbance of this axis can lead to autoimmune diseases because AT1R activation triggers diverse signaling cascades involved in inflammation, fibrosis and tissue remodeling. The counter-regulatory angiotensin-converting enzyme 2 (ACE2) axis involving Ang receptors and Ang(1–7) has also been implicated in regulating immune responses and tissue homeostasis within the skin microenvironment. In fact, as ACE2 is the entrance point for severe acute respiratory syndrome coronavirus 2 (SARS-CoV-2), both infection and vaccination have been associated with worsening inflammatory skin conditions, including psoriasis and atopic dermatitis (AD) [2].

Adverse effects due to ACE inhibitors are frequently observed on the skin [3]. Approximately 10%–60% of negative drug reactions from diuretics, beta-blocking agents, and antihypertensive drugs are dermatological [4] and include life-threatening angioedema, pruritus, bullous eruptions, urticaria, other generalized rashes, photosensitivity and hair loss [3]. Beta blockers are significantly associated with psoriasis, lichen planus, subacute cutaneous lupus erythematosus, and an increased risk of skin cancer [5]. Psoriasis is among the main adverse cutaneous effects of ACE inhibitors, possibly due to the accumulation of vasoactive mediators, prevention of binding of Ang II to AT1 and AT2 receptors and the presence of circulating antibodies [3, 6]. ACE also degrades bradykinin (BK), substance P, enkephalin and other important peptide hormones [3]. Treatments targeting specific components of the RAS could ameliorate inflammatory responses and lessen disease manifestations, but considerable research is still needed.

Some studies have shown increased serum ACE activity in psoriasis patients [7–9], whereas other researchers have not reported that it is a typical pattern of the disease [10, 11]. It was also reported that therapy decreased serum ACE activity to normal values [8, 12, 13]. However, the investigations varied considerably with regard to patient selection and purpose considering co-factors such as atherosclerosis, sarcoidosis and cardiovascular disease; thus, the relationship between ACE levels and psoriasis was unclear. In AD patients, serum ACE levels vary significantly but are centered around the mean value observed in healthy controls [14].

**Fig. 1:**
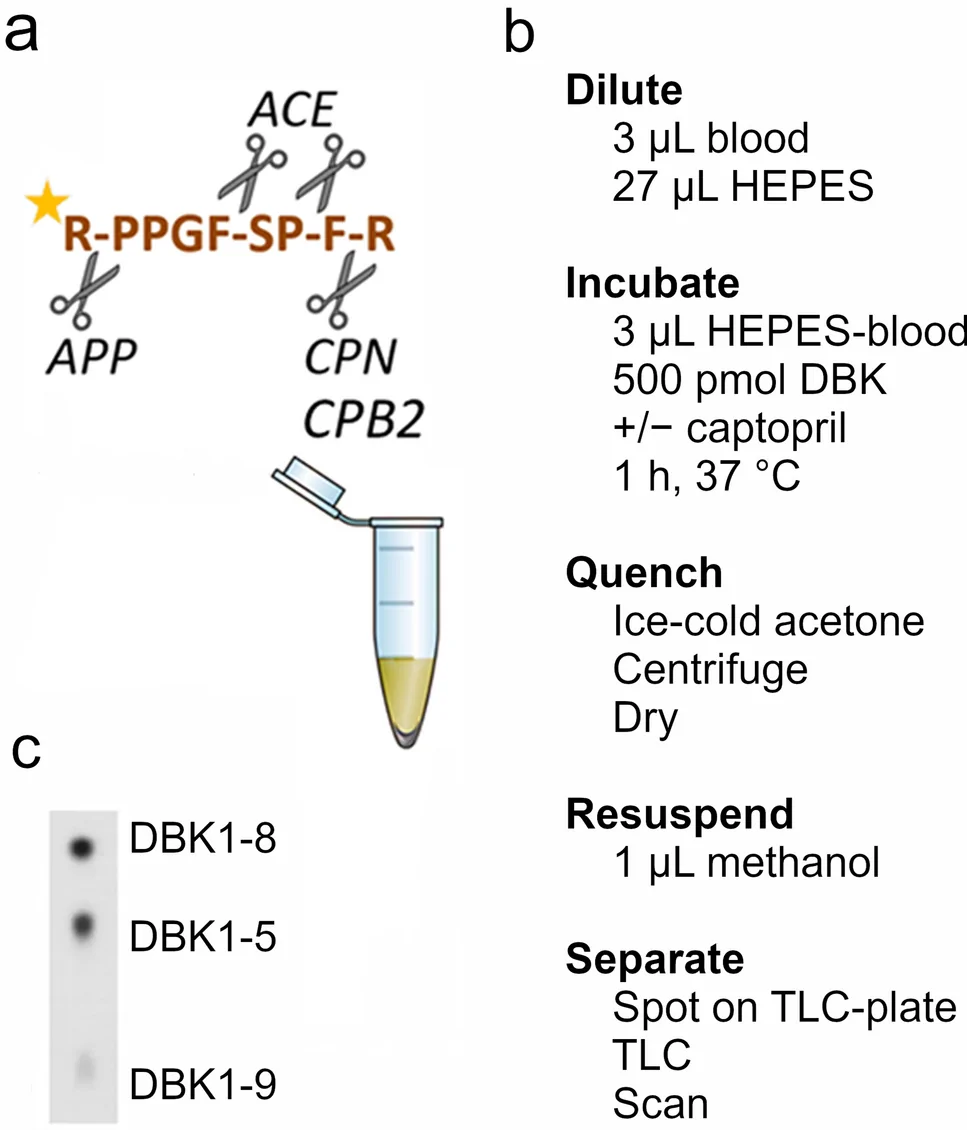
Summary of the neuropeptide reporter assay (NRA). (**a**) Bradykinin (BK) sequence and enzyme cleavage sites. Important are the sites for angiotensin-converting enzyme (ACE) and basic carboxypeptidase (CPN/CPB2) cleavage, leading to the formation of DBK1–5 (RPPGF) and DBK1–8 (RPPGFSPF), respectively, from dabsylated BK (DBK). (**b**) NRA workflow: sample preparation and thin-layer chromatography (TLC) separation of incubation products. (**c**) Exemplary TLC result showing spots for residual starting material and both enzymatic fragments of DBK. APP, aminopeptidase P; the peptide is labeled at the N-terminus (★)

In contrast to traditional ACE measurements, which are typically based on enzyme-linked immunosorbent assays (ELISAs), we used the vasoactive synthetic peptide BK (Fig. 1a) in its dabsylated form (DBK) as a reporter substance to determine ACE activity in blood [15]. In earlier work with this neuropeptide reporter assay (NRA; Fig. 1), we reported that serum ACE activity is compromised in patients with complex regional pain syndrome, a neurological condition that causes long-lasting pain, edema, and inflammation in the extremities [16]. In coronavirus disease 2019, the level of the DBK cleavage product generated by ACE is increased and correlated with clinical heart and liver parameters [17]. In both instances, the RAS was dysregulated, albeit for different reasons.

Here, we measured the degradation capacity for DBK of enzymes in blood obtained from patients with psoriasis vulgaris and AD. Both diseases are characterized by chronic inflammatory skin conditions. Psoriasis patients exhibit an overactive immune response with heterogeneous clinical features, including cutaneous and systemic manifestations [18]. In AD, disrupted skin barrier function causes dry, itchy, and inflamed skin [19]. With the NRA, the cleavage of DBK (also called DBK1–9 for the length of the peptide) by ACE and basic carboxypeptidases (carboxypeptidase N [CPN], CPB2) was determined using thin-layer chromatography (TLC) of fragments DBK1–5 and DBK1–8 (Fig. 1). The CPN is a representative of the kallikrein-kinin system (KKS), which is interwoven with the RAS by the ACE. The ACE contribution in our experiments was evaluated with and without the ACE-specific inhibitor captopril.

## Materials and Methods

### Patients

The pilot study was approved by the Ethics Committee of the University of Münster (2019-383-f-S, May 27, 2019) and was in agreement with the Helsinki Declaration of 1975 as last revised in 2024 (75th World Medical Association General Assembly, Helsinki, Finland). The patients provided written informed consent for the publication of their case details.

This pilot study attempted to exclude as many cofactors to target diseases as possible (e.g., age-related diseases) and thus focused only on younger male patients at our clinic. Females were also excluded to avoid hormonal influences [20]. The clinical characteristics of the included probands at the time of blood sampling are shown in Table 1. All clinical diagnoses were made by dermatologists at the Department of Dermatology of our clinic. Patients with symptoms of other illnesses were excluded. The control samples were obtained from five healthy male volunteers from the staff of our clinic (aged 25–37 years) who experienced no symptoms of any disease and were not overweight. None of the patients or healthy control individuals took antihypertensive medication, such as ACE inhibitors.

**Table 1:** Clinical characteristics of the patient groups and controls (all male). The means (range) are indicated

Variables	Control group (n = 5)	Atopic dermatitis (n = 5)	Psoriasis vulgaris (n = 4)
Age (years)	28.8 (25–37)	25.8 (19–40)	30 (23–40)
Disease duration (years)	–	17.3 (10–23)	8 (1–14)
PASI	–	–	15.3 (9–22)
SCORAD	–	55.8 (49–66)	–
DLQI	–	–	20 (5–26)
NRS	–	7 (4–9)	
Comorbidities^*^	–	Allergic asthma (4/5), allergic rhinitis (4/5), food allergy (1/5)	Obesity^#^ (1)
Arterial hypertension	–	–	–
Current medication^*^	–	TCS^6^ (5/5), TCI^7^ (3/5), antihistamines (4/5), Dupilumab (1/5)	TCS^6^ (3/4), vitamin D analogs (2/4)

^*^Numbers in brackets indicate the number of patients of the total n

^#^Only one patient was obese (BMI > 30.5 kg/m²)

*PASI* Psoriasis Area and Severity Index, *SCORAD* Severity Scoring of Atopic Dermatitis, *DLQI* Dermatology Life Quality Index, *NRS* Numeric Rating Scale of Pruritus, *TCS* topical corticosteroids, *TCI* topical calcineurin inhibitors

### Methods

The protease activity of individual samples was determined by TLC following the incubation of blood with DBK. Whole venous blood was drawn and immediately frozen (−20 °C). It was later aliquoted, frozen, and thawed as needed. Experiments were performed in triplicate as described previously (for development and validation of the NRA, see [15, 21], Fig. 1b) with slight changes. Briefly, whole blood aliquots (3 μL) were diluted with 27 μL of 2-(4-(2-hydroxyethyl)-1-piperazinyl)-ethansulfonic acid (HEPES) buffer. Three microliters of these solutions were added to dry DBK (500 pmol) and incubated for 1 h at 37 °C at 800 rpm. This timepoint was chosen because of its robustness; in healthy subjects, DBK degradation to DBK1–8 and DBK1–5 was nearly complete (Fig. 1c). The reaction was stopped with 60 μL of ice-cold acetone, and the proteins were precipitated overnight at −20 °C. The samples were centrifuged, and the supernatant was dried and redissolved in 1 μL of methanol for TLC. For inhibition experiments, captopril (1 mg/mL, 5 μL) was added to dry DBK, which was subsequently allowed to dry before it was incubated with blood. TLC images were analyzed using JustTLC (Sweday, Sweden). To account for sample loss during sample preparation and plate-to-plate variation, the relative values of each spot were calculated by dividing the individual intensity by the summed intensity of all the spots of the sample. Statistical analyses (tests for normality and independent *t* tests) were performed using IBM SPSS Statistics 29.0.0. (241). The data are provided in the Supplementary Materials.

## Results and Discussion

**Fig. 2:**
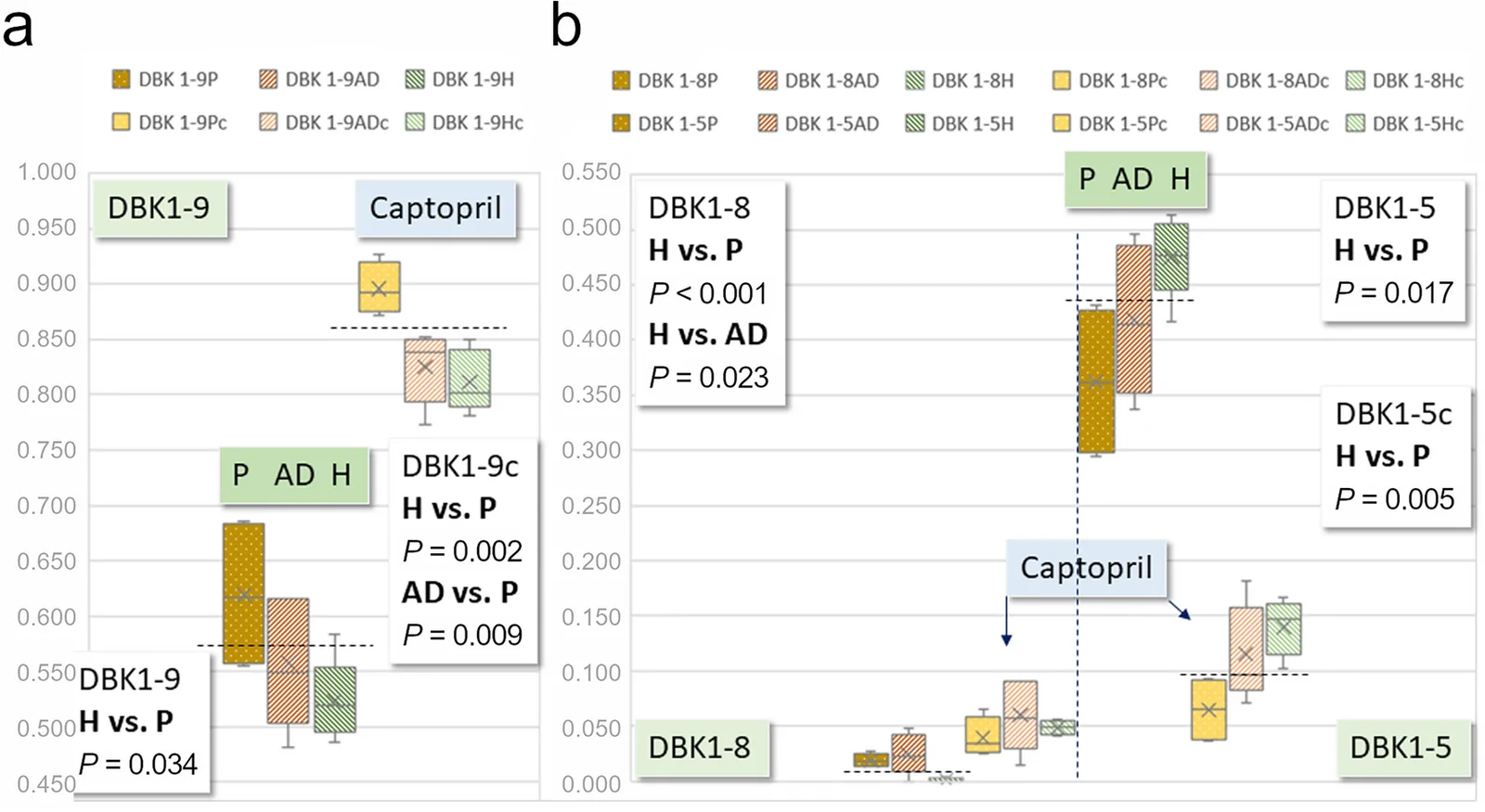
Relative spot intensities for dabsylated bradykinin (DBK) and its degradation fragments in whole blood for patients (y-axis) with psoriasis (P, n = 4), atopic dermatitis (AD, n = 5) and healthy volunteers (H, n = 5). The results for DBK1–9 (a) and its enzymatic fragments DBK1–8 and DBK1–5 (b) were significantly different in P vs. H, as indicated by the *P* values. For AD, only DBK1–8 differed significantly from the value measured in the blood of healthy controls. The addition of captopril (values indicated by “c”) considerably reduced the degradation of DBK in all groups, but the greatest effect was observed for P. Independent *t* tests were performed with SPSS. For data and statistical calculations, see the Supplementary Materials. Note that the power of study is limited

Approximately one-tenth of the volume of whole blood had the same DBK degradation capacity as serum but showed much greater ACE activity. In serum measurements, the dominant fragment after 1 h of incubation with DBK was DBK1–8, whereas in whole blood from healthy male donors, it was DBK1–5. Whole blood was thus more suitable for studying ACEs. The results are shown in Fig. 2. In psoriasis patients, the degradation capacity of DBK was significantly lower (*P* = 0.034) and that of DBK1–5 was lower (*P* = 0.017), but more DBK1–8 (*P* < 0.001) was formed. In AD patients, the results were not as dramatic: while the data spread was increased in disease patients, only DBK1–8 was significantly different from that in healthy controls (*P* = 0.023). The degradation capacity for DBK in AD patients was better than that in psoriasis patients and close to that in controls. In all cases, the amount of digested DBK matched the sum of the amounts of generated DBK1–5 and DBK1–8. The loss of DBK1–9 was inversely correlated with the increase in DBK1–5 (i.e., ACE activity) in all samples (Pearson’s *r* =  −0.981; *P* < 0.001). Captopril almost abolished DBK cleavage in psoriasis patients, in contrast to that in healthy and AD subjects, where 80%–85% of undigested DBK remained (psoriasis vs. healthy or AD patients: *P* = 0.002, 0.009). The inhibited amount of ACE was approximately the same in all the samples and matched the decrease in DBK1–5 formation. DBK1–9 degradation and DBK1–5 formation (i.e., ACE activity) without and with inhibition were, as expected, correlated (Pearson’s *r* = 0.553; *P* = 0.040, 0.750, 0.002). With respect to inhibition, the loss of DBK1–9 was also inversely correlated with the increase in DBK1–5 (Pearson’s *r* =  −0.883; *P* < 0.001). The DBK fragment generated by basic carboxypeptidases (DBK1–8) was measured at the detection limit and did not increase with ACE inhibition to an extent that matched the loss of DBK1–5. The greatest increase was detected in samples from healthy subjects, indicating that more readily available CPN/CPB2 activity is needed for DBK digestion. Notably, although the enzymes compete for the substrate DBK, in the present experiments in whole blood, in contrast to serum, DBK1–5 was the main product.

Thus, compared with AD, ACE seems to have a greater influence on psoriasis, as reflected in the literature (see above). We could, however, not confirm the increased ACE activity in the circulation reported by other authors in psoriasis patients [7–9]. Reasons include the use of different detection methods (ELISA vs. NRA) and the size and composition of the patient cohorts. We need to stress that our choice of samples regarding the age and sex of the patients (younger males) constitutes a serious selection bias, which has a profound impact on the generalizability of the study conclusions. It was necessary to evaluate the usability of the NRA for this type of disease first before a larger multicenter investigation could be attempted, especially because we had learned in an earlier community study of hundreds of serum samples from healthy volunteers that the assay is highly sensitive to preanalytical factors [22]. The quality of phlebotomy and the skill of the study nurses are major factors in any analytical workflow, and the NRA was able to select low-quality sera on the basis of their reduced enzyme activity even among the nonhemolytic samples [22]. Of course, future larger studies will include female probands, particularly because both psoriasis and AD are highly prevalent among women. Another selection bias resulted from our choice of healthy volunteers who were recruited from the clinical staff of our institution.

We also need to remind the reader that we worked with whole blood rather than serum, which is normally used in clinical laboratories. Our initial goal was to use capillary blood from inflamed sites to take advantage of the ability of the NRA to handle whole blood [21] in quick tests [23], but our proposed sampling method using calibrated capillaries was not practical in daily clinical practice; thus, we worked with venous blood drawn from the circulation initially. Clearly, differences between the enzyme activities in serum and blood are expected, if only the presence of clotting factors is mentioned. In normal lab work, serum is the matrix of choice because it presents a stable liquid essence with a reduced danger of clogging analytical instrumentation. Thus, the comparability of our results to those of other researchers is limited with respect to the sample matrix. Finally, disease severity in individual patients may have had an impact on the outcomes of the experiments.

## Conclusions

We performed a small exploratory study testing the use of DBK digestion by blood proteases in the treatment of dermatological inflammatory diseases and reported a significantly reduced capacity for cleaving DBK in the blood of young male psoriasis patients (n = 4) in contrast to AD patients (n = 5) and controls. The detected activity was due mainly to ACE and could be inhibited by captopril. Generally, in patient samples, the variation in the measured values was greater for serum ACE levels in AD patients than in healthy controls, as observed by others [14]. Our experiments had limited power but could not confirm the increased ACE activity in the circulation reported in psoriasis patients [7–9], likely because of different study designs (size and composition of the cohort, choice of sample matrix and detection method). We were careful not to include samples of female patients in this pilot phase because RAS enzyme activities may vary with the female cycle [20]. Obviously, our results cannot be generalized to women, and the following larger studies will represent the general population more adequately with respect to age. To avoid the influence of age-dependent diseases such as hypertension, which could be treated with ACE inhibitors, we focused on younger men. This was a pilot and feasibility study on a small number of patients and controls. The heterogeneity of the two patient cohorts, e.g., with regard to disease duration, severity and medication, and the controls in comparison may have influenced the levels of the measured cleavage products.

Despite these limitations, our pilot study certainly questions the current view of the role of ACE in psoriasis. We have learned that the robust and cost-effective NRA allows monitoring of the activity of basic KKS/RAS enzymes in dermatology research and are now ready to verify and extend the results of this preliminary investigation in a larger cohort. In particular, whether ACE can be a marker of disease severity in psoriasis needs to be clarified, as suggested by other authors [9]. To that end, both ELISA and NRA-based methods can be standardized for a large-scale population study encompassing both sexes and a wide age range.

## Supplementary Information

Below is the link to the electronic supplementary material.

Supplementary file1 (XLSX 31 KB)

## Data Availability

The data presented in this study are available in the supplementary materials.
